# Supplementary material for: Patient-reported pain and physical health for acupuncture and chiropractic care delivered by Veterans Affairs versus community providers
Source: PLoS One. 2024 May 15;19(5):e0303651. doi: 10.1371/journal.pone.0303651 (PMC11095679; doi:10.1371/journal.pone.0303651)
Supplement: S1 Table — (DOCX) [file pone.0303651.s001.docx]

|  |  | Coefficient^a^ (VA relative to community) | p | Coefficient^b^ (VA relative to community) | p |
| --- | --- | --- | --- | --- | --- |
| Acupuncture | Pain | -0.095 | 0.567 | -0.222 | 0.270 |
|  | Physical health | 0.653 | 0.243 | 0.500 | 0.463 |
| Chiropractic | Pain | -0.196 | 0.282 | -0.203 | 0.314 |
|  | Physical health | 0.762 | 0.180 | 0.775 | 0.224 |

^a^Controlling for baseline outcome score, total number of visits during study period only
^b^Controlling for baseline outcome score, total number of visits during study period, demographics
