# Supplementary material for: Patient-reported pain and physical health for acupuncture and chiropractic care delivered by Veterans Affairs versus community providers
Source: PLoS One. 2024 May 15;19(5):e0303651. doi: 10.1371/journal.pone.0303651 (PMC11095679; doi:10.1371/journal.pone.0303651)
Supplement: Attachment — Submitted filename: Response to Reviewers.docx [file pone.0303651.s002.docx]

Response to Reviewers

When submitting your revision, we need you to address these additional requirements.

1. Please ensure that your manuscript meets PLOS ONE's style requirements, including those for file naming. The PLOS ONE style templates can be found at

<https://journals.plos.org/plosone/s/file?id=wjVg/PLOSOne_formatting_sample_main_body.pdf> and

<https://journals.plos.org/plosone/s/file?id=ba62/PLOSOne_formatting_sample_title_authors_affiliations.pdf>

**Complete**

2. In the online submission form you indicate that your data is not available for proprietary reasons and have provided a contact point for accessing this data. Please note that your current contact point is a co-author on this manuscript. According to our Data Policy, the contact point must not be an author on the manuscript and must be an institutional contact, ideally not an individual. Please revise your data statement to a non-author institutional point of contact, such as a data access or ethics committee, and send this to us via return email. Please also include contact information for the third party organization, and please include the full citation of where the data can be found.

**I am working on obtaining this and will send when I have this information**

3. We notice that your supplementary table (Appendix Table) is included in the manuscript file. Please remove them and upload them with the file type 'Supporting Information'. Please ensure that each Supporting Information file has a legend listed in the manuscript after the references list.

**Complete**

4. Please review your reference list to ensure that it is complete and correct. If you have cited papers that have been retracted, please include the rationale for doing so in the manuscript text, or remove these references and replace them with relevant current references. Any changes to the reference list should be mentioned in the rebuttal letter that accompanies your revised manuscript. If you need to cite a retracted article, indicate the article’s retracted status in the References list and also include a citation and full reference for the retraction notice.

**Complete**

[Note: HTML markup is below. Please do not edit.]

Reviewers' comments:

Reviewer's Responses to Questions

**Comments to the Author**

1. Is the manuscript technically sound, and do the data support the conclusions?

The manuscript must describe a technically sound piece of scientific research with data that supports the conclusions. Experiments must have been conducted rigorously, with appropriate controls, replication, and sample sizes. The conclusions must be drawn appropriately based on the data presented.

Reviewer #1: Yes

Reviewer #2: Yes

2. Has the statistical analysis been performed appropriately and rigorously?

Reviewer #1: Yes

Reviewer #2: Yes

3. Have the authors made all data underlying the findings in their manuscript fully available?

The [PLOS Data policy](http://secure-web.cisco.com/1mbhxDJJ0c4v2K8lzU-CyDtGrkdybAehpNuMzI5pG-mMfn62JguLA6KhPv_IipAC0p3vDWn7UwXUdtzznyuujLlwk4lX5vAYxJE_qK8TRZeTMP1ObRKaaN1jaB0bYEYEP_vOumN1n4ELsOGgqyJdTR9kfEaX_nEXUP1R5RYpLZv5T2N-aiof6ESkXX1z-9WtF7vb3WXzcVVJcdeob6JC2P2Zin-cy0ne6YIdBWOmd_9VILTOyDobKz5Cs1z8esXhLMu8lPg6A0-_aHnrOUwnfGeoep4apcYXh0IruQZxA3rXZxt5xrtvE06tyr8NnZBnudll9Jj09eP5qzE_8VtPnHUQFU7sutjz4yUQJinX8Qg9AyN-3evZek7NfzFkRJNP8/http%3A%2F%2Fwww.plosone.org%2Fstatic%2Fpolicies.action%23sharing) requires authors to make all data underlying the findings described in their manuscript fully available without restriction, with rare exception (please refer to the Data Availability Statement in the manuscript PDF file). The data should be provided as part of the manuscript or its supporting information, or deposited to a public repository. For example, in addition to summary statistics, the data points behind means, medians and variance measures should be available. If there are restrictions on publicly sharing data—e.g. participant privacy or use of data from a third party—those must be specified.

Reviewer #1: No

Reviewer #2: Yes

4. Is the manuscript presented in an intelligible fashion and written in standard English?

PLOS ONE does not copyedit accepted manuscripts, so the language in submitted articles must be clear, correct, and unambiguous. Any typographical or grammatical errors should be corrected at revision, so please note any specific errors here.

Reviewer #1: Yes

Reviewer #2: Yes

5. Review Comments to the Author

Please use the space provided to explain your answers to the questions above. You may also include additional comments for the author, including concerns about dual publication, research ethics, or publication ethics. (Please upload your review as an attachment if it exceeds 20,000 characters)

Reviewer #1: Thank you for inviting me to review this paper. This manuscript is well-written, and the authors provide a very good introduction with relevant background information on the topic and rationale for their study. The methods are clear, and the results are well-presented. I have only a few minor edits and suggestions for the authors to consider.

Abstract, Materials and Methods – The authors could consider adding the word “in” before “2018-2019”.

Methods, Analysis section (top of p. 9) – I’d recommend adding the word “examine” before “differences”.

Results, Acupuncture – There were non-statistically significant differences on categories of race between VA and community acupuncture patients. Statistically significant differences were observed between groups on age and region of residence.

Limitations – The authors could consider moving this section to the end of the Discussion. I’d also recommend considering changing the word “observe” to “observed” (p. 10), and the word “do” to “did” and “be” to “have been”, and “can” to “could” and “are” to “were”, and “have not sought” to “did not seek” (p. 11, sentence starting with, “We also do not control for …”).

Discussion, p. 12 – I’d recommend the authors consider revising the sentence starting with, “While most studies comparing VA and non-VA …” to something like, “While most studies comparing VA and non-VA care examine inpatient and emergency care, prior studies of outpatient care have found that VA outpatient providers provide better or similar patient experiences to community outpatient providers.29,30”

**Thanks for these suggestions, we have implemented all of them.**

Reviewer #2: Thank you for the invitation to evaluate “Patient-reported pain and physical health for acupuncture and chiropractic care delivered by Veterans Affairs versus community providers. The authors a report on quality improvement project to evaluate outcome variation (pain and physical function) for Veterans at the 18 VA Whole Health Flagship sites who received at least 4 visits of VA or-VA acupuncture or chiropractic care. The methods and statistical approach are appropriate for this investigation. The findings of this study add valuable preliminary insight to the differences in care outcomes when Veterans are rendered care at VA or community. I applaud the authors for their efforts.

As there are no line numbers in the proof, I will reference section and paragraph number when possible.

Several considerations for the authors:

INTRODUCTION:
Paragraph 2: Additional citation for on-station chiropractic services could be considered:
Halloran SM, Coleman BC, Kawecki T, Long CR, Goertz C, Lisi AJ. Characteristics and Practice Patterns of U.S. Veterans Health Administration Doctors of Chiropractic: A Cross-sectional Survey. J Manipulative Physiol Ther. 2021 Sep;44(7):535-545. doi: 10.1016/j.jmpt.2021.12.005.

Lisi AJ, Brandt CA. Trends in the Use and Characteristics of Chiropractic Services in the Department of Veterans Affairs. Journal of Manipulative and Physiological Therapeutics. 2016;39(5):381-386. doi:10.1016/j.jmpt.2016.04.005
**Thanks for these suggested citations, we have added them.**

Paragraph 3, sentence 1: “Comparing quality of care provided....” Is this citable? What is the supporting evidence?
**We have added a citation to recent congressional testimony on this issue.**

Additional context for the development and employment of the workforce to deliver acupuncture and chiropractic is relevant as these services are recent additions to VHA – acupuncture more so than chiropractic. Please revise to enhance the introduction. I have included supporting citations related to this workforce:

Kligler B, Niemtzow RC, Drake DF, Ezeji-Okoye SC, Lee RA, Olson J, Reddy KP. The Current State of Integrative Medicine Within the U.S. Department of Veterans Affairs. Med Acupunct. 2018 Oct 1;30(5):230-234. doi: 10.1089/acu.2018.29087-rtl. Epub 2018 Oct 15.

Olson J, Kligler B. Society for Acupuncture Research Turning Point: Acupuncture in the Veterans Health Administration. J Altern Complement Med. 2021 Jul;27(7):527-530. doi: 10.1089/acm.2021.0194.

Olson JL. Licensed Acupuncturists Join the Veterans Health Administration. Med Acupunct. 2018 Oct 1;30(5):248-251. doi: 10.1089/acu.2018.1298. Epub 2018 Oct 15. PMID: 30377460; PMCID: PMC6205763.

Lisi AJ, Khorsan R, Smith MM, Mittman BS. Variations in the Implementation and Characteristics of Chiropractic Services in VA: Medical Care. 2014;52:S97-S104. doi:10.1097/MLR.0000000000000235

Lisi AJ, Goertz C, Lawrence DJ, Satyanarayana P. Characteristics of Veterans Health Administration chiropractors and chiropractic clinics. JRRD. 2009;46(8):997. doi:10.1682/JRRD.2009.01.0002
**Thanks for these suggested citations, we have added the newer citations (post-2017, when the Whole Health demonstration project started) since the older characteristics articles may be outdated.**

METHODS:
Was this manuscript reporting consistent with study design guidelines? For example, SQUIRE (quality improvement) or STROBE (cohort)?
**Yes, we’ve adhered to the STROBE guidelines.**

Paragraph 2: Survey Sampling Approach:
Second sentence: For VA’s Whole Health demonstration project, please consider citing the initiative.
**Complete**

While a dose of 4 visits at least seems reasonable from a course of care where Veterans may be lost to follow-up or achieve outcome. Is there a source to support the rationale for dose selection?
**This has come up a lot and we could not identify literature out there to support dosing for either of these modalities, so this was based on expert opinion of senior members of the research team. We have added that this was based on expert opinion.**

RESULTS:
Page 9, Acupuncture: Race is noted as significant between VA and community acupuncture patients. In contrast, Table 1 fails to bold ‘race’ for acupuncture indicating in current form the difference is not significant. Please revise for continuity.
**Thanks for catching this, complete**

Regarding limitations, is there something unmeasured related to the 18 Whole Health Pilot VAs?

There was one facility per VISN (VA regional network) – we added a phrase about this in the paper. It’s not totally clear how the sites were selected, but it wasn’t random. We changed the last sentence of the limitations to “Lastly, these results may or may not generalize to the population of Veterans with chronic pain, as this study only includes Veterans who received acupuncture or chiropractic care and were patients at the VA facilities that were part of the VA demonstration project.”

What was the representation of Veterans across the 18 medical centers? How do I know that I am on looking at the comparison of Veterans at a single site and not the suggested cross-section of the 18 VAs. Is there clarity to the distribution? Perhaps this was lost with the data de-linking step. If this is available, please consider reporting.

**This is not available, but based on the geographic distribution of the data in Table 1, at least one site from each of the nine regions is represented, so there must be at least nine distinct sites.**

DISCUSSION
Paragraph 1 of the introduction frames interest in determining the advantages or disadvantages of referring to one care setting or another – VA vs. non-VA. Please consider revisions to the Discussion to draw on this notion. Considerations beyond the scope of this study, but worth commenting on:

1) Do on station services compared to non-VA services have increased likelihood of care coordination and utilization for other VA services thus enhancing use of VA care.

Thomas ER, Zeliadt SB, Coggeshall S, et al. Does Offering Battlefield Acupuncture Lead to Subsequent Use of Traditional Acupuncture? Medical Care. 2020;58:S108-S115.

Etingen, B., Smith, B.M., Zeliadt, S.B. et al. VHA Whole Health Services and Complementary and Integrative Health Therapies: a Gateway to Evidence-Based Mental Health Treatment. J GEN INTERN MED 38, 3144–3151 (2023).

**This is a great point, we have added this sentence and citation to the discussion: There may be other distal benefits to having such care provided within VA, such as coordination and linkages with other kinds of care [cites].**

2) Do divergent downstream care cascades occur between those Veterans who attended VA or non-VA care? Some of these are guideline discordant and commonly low-value health services: imaging, specialist referrals, and opioids prescriptions.
3) Does divergent health care utilization costs result from use of VA vs non-VA care?
**These are also both good points, we have added a sentence related to this in the discussion: “The use of such care within or outside the VA could also potentially result in different care utilization cascades downstream, such as for imaging, referrals to specialists, or prescriptions for opioids, which has important implications for costs and outcomes.”**

4) Finally, overall cost episode and cost per visit is worth mentioning. Outcomes are similar at VA and non-VA after adjusting for demographics, visit count. However, what this does tell me is on-station VA care is more efficient in overall cost. Both policy makers, administrators, referring physicians, and patients should recognize the value of cost-effective care when having to choose where and when to ‘buy’ the ‘outcome’ when choosing VA or non-VA care.
**We don’t have information or data on the costs of care or reimbursement; it could be that VA compensates community providers much less than providers in-house. I’ve revised the sentence about this to the following to be more clear: “It is especially notable that differences in outcomes were not observed while utilization was higher for patients using community providers, which could mean that VA care is more efficient if the cost per visit is the same.”**

Table 1: Consider including n for each ‘n’ in addition to ‘%’
**This table is already pretty busy and the n’s are calculable from the headers, so I would prefer to leave as is.**

6. PLOS authors have the option to publish the peer review history of their article ([what does this mean?](https://secure-web.cisco.com/1OfLwCRIEP_KtgrMZ-tYVAuNq3ozl28E7Rqr4bovjb6vP9EfAcVPf1vH7gOcydrFbQjJL_zpost_cOBRynRr9ANrtBOCRZpqoR8Vp5tdZwx5ejRVmNy1pqUpplTC6lxQghcSa4Mur05Zcnc-VEcw1dREl9Ee7YdPX1WvfvNSHfqTw0mZPEXieooC5t5Yt2Zd6FsMXPeaQE7SOKEH_9U2CX7Eeu0zPRaRfG3iaccTsTH9oO5P9IBegHx6CVr0PaSRvjBkCghwMVoFMPrmw8QbGQ8BOcoxS6kBGMn4Gc785VcfYmtSm_3p05_KVbd0SxyvwgZOhNQJgylm4g2_1eVULHx7IJrD8I8lT4cjKsYhhAe_SDSJmuns40KetVcc9yfDo/https%3A%2F%2Fjournals.plos.org%2Fplosone%2Fs%2Feditorial-and-peer-review-process%23loc-peer-review-history)). If published, this will include your full peer review and any attached files.


If you choose “no”, your identity will remain anonymous but your review may still be made public.


**Do you want your identity to be public for this peer review?** For information about this choice, including consent withdrawal, please see our [Privacy Policy](https://secure-web.cisco.com/1V-ty5q7_mdB04TKanoxiem4SZIQ77kgBP40-uDcombAH_HDGBddBQDpyOeOp2DYigU_X2reG3QpXPH4AlyjEUGG7cy_3AHZe24jJVqzwGScYiXPI4YWdXItL_tFa3GrMtmqYurCTJxEtH9Fsgi4Zi8VH6TxVHfO1qBbc2m2NmvNjXbLf2ieEeqrIvF0rmEApo_BetrKVNajwVhabyYnO8TYpFuNyItUvWpesYXudVNX2iUd8HF7yGT5b2a7BM-3jG5tFTytIu2-yZDhnnUFIkKuutXvdi8ef3bH_4BbeTsjOCmkunYd5POZax1PmxaGWAbOMhYYaHxmQkpSbK-y7WiUwDiE0liOeXCxwzC2EpSLMCWHEGoTdVj7orxSD2Si0/https%3A%2F%2Fwww.plos.org%2Fprivacy-policy).

Reviewer #1: No

Reviewer #2: **Yes:** Zachary Cupler

[NOTE: If reviewer comments were submitted as an attachment file, they will be attached to this email and accessible via the submission site. Please log into your account, locate the manuscript record, and check for the action link "View Attachments". If this link does not appear, there are no attachment files.]

While revising your submission, please upload your figure files to the Preflight Analysis and Conversion Engine (PACE) digital diagnostic tool, [https://pacev2.apexcovantage.com/](https://secure-web.cisco.com/1D3PJ0PZhUsrOoe4c8wkuslxgvFpxB9KgNkmoSW8ZHwDJw7lDN50StJ2QtPAKGKrP4xGFQNE6TKdYt14LDY8iUeBaumEPZLN8FezJA2S34Y86DreKh7g_eFy1pO85-BaESOIyQx2TFIbl8iqYVNXRnpx6ADp71Kt9pzoorOBqWoipoIkNwoBuTq6IZfK_atH98NQP2deQnZCcBP4chpy3RwXUAOLgya-LNejpbJUWRZ3oAAZslz16i8kLVFqWPCK7Mr-_gpBtjO5S3tDBbm6fBNURD5l2FsX0YCLvxBD6hMcno4OOkqRMYT8WeL3CEpYqK5pzfqa7iaQApaH8j0YsiMPEFLvT3nuo1Etb5Ko4dUNBmEYIJLbI_fLJd4tmIJDv/https%3A%2F%2Fpacev2.apexcovantage.com%2F). PACE helps ensure that figures meet PLOS requirements. To use PACE, you must first register as a user. Registration is free. Then, login and navigate to the UPLOAD tab, where you will find detailed instructions on how to use the tool. If you encounter any issues or have any questions when using PACE, please email PLOS at [figures@plos.org](mailto:figures@plos.org). Please note that Supporting Information files do not need this step.
